# Role of the ISR-ATF4 pathway and its cross talk with Nrf2 in mitochondrial quality control

**DOI:** 10.3164/jcbn.18-37

**Published:** 2018-09-15

**Authors:** Shuya Kasai, Hiromi Yamazaki, Kunikazu Tanji, Máté János Engler, Tomoh Matsumiya, Ken Itoh

**Affiliations:** 1 Department of Stress Response Science, Center for Advanced Medical Research, Hirosaki University Graduate School of Medicine, 5 Zaifu-cho, Hirosaki 036-8562, Japan; 2 Department of Neuropathology, Hirosaki University Graduate School of Medicine, 5 Zaifu-cho, Hirosaki 036-8562, Japan; 3 Department of Vascular Biology, Hirosaki University Graduate School of Medicine, 5 Zaifu-cho, Hirosaki 036-8562, Japan

**Keywords:** ATF4, Nrf2, mitochondria, GCN2

## Abstract

Recent investigations have clarified the importance of mitochondria in various age-related degenerative diseases, including late-onset Alzheimer’s disease and Parkinson’s disease. Although mitochondrial disturbances can be involved in every step of disease progression, several observations have demonstrated that a subtle mitochondrial functional disturbance is observed preceding the actual appearance of pathophysiological alterations and can be the target of early therapeutic intervention. The signals from damaged mitochondria are transferred to the nucleus, leading to the altered expression of nuclear-encoded genes, which includes mitochondrial proteins (i.e., mitochondrial retrograde signaling). Mitochondrial retrograde signaling improves mitochondrial perturbation (i.e., mitohormesis) and is considered a homeostatic stress response against intrinsic (ex. aging or pathological mutations) and extrinsic (ex. chemicals and pathogens) stimuli. There are several branches of the mitochondrial retrograde signaling, including mitochondrial unfolded protein response (UPR^MT^), but recent observations increasingly show the importance of the ISR-ATF4 pathway in mitochondrial retrograde signaling. Furthermore, Nrf2, a master regulator of the oxidative stress response, interacts with ATF4 and cooperatively upregulates a battery of antioxidant and antiapoptotic genes while repressing the ATF4-mediated proapoptotic gene, CHOP. In this review article, we summarized the upstream and downstream mechanisms of ATF4 activation during mitochondrial stresses and disturbances and discuss therapeutic intervention against degenerative diseases by using Nrf2 activators.

## Introduction

Since the uptake of α-proteobacteria by archaea approximately 1.5 billion years ago, communication between the two entities has crucial importance in the generation and development of eukaryotes.^(1)^ As α-proteobacteria are transformed into the current mitochondria, the energy generation of the eukaryotes is increasingly confined to the mitochondria by oxidative phosphorylation (OxPhos). In the context of current eukaryotes, mito-nuclear communication is important, particularly because OxPhos proteins in the mitochondria are mosaics of nuclear- and mitochondrial-encoded proteins, except for complex II, and mitochondrial-encoded proteins act as a core of OxPhos protein assembly and have to be strictly matched for efficient OxPhos.^(2)^ Originally mitochondria may utilize locally generated reactive oxygen species (ROS) as a signal within the mitochondria in response to the increased energy demand to change the content and the assembly of mitochondrial electron transport chain (mETC) or to increase mitochondrial biogenesis (i.e., reactive biogenesis).^(1,3)^ As yet undetermined signals orchestrate the coordinated biosynthesis of mitochondrial and nuclear-encoded proteins to maximize the efficiency of OxPhos. A recent report in yeast demonstrated that the regulatory mechanism of translation orchestrates the balanced synthesis of mitochondrial- and nuclear-encoded OxPhos proteins during the energy shift from glycolysis to OxPhos.^(4,5)^

**Fig. 1:**
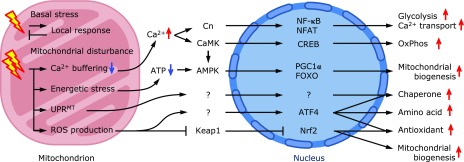
Mitochondria-to-nuclear retrograde signaling pathways in mammals. Mitochondrial respiration produces intrinsic ROS, which is eliminated by endogenous antioxidants. Various stresses produce deleterious excess ROS and cause mitochondrial dysfunction that results in the declined synthesis of ATP and mitochondrial metabolites, loss of mitochondrial membrane potential and Ca^2+^ release, accumulation of unfolded protein and further production of ROS. Energetic stress activates AMPK and induces expression of PGC1α- and FOXO-regulated genes that are involved in mitochondrial biogenesis and mtDNA replication.^(107,108)^ Increased cytoplasmic Ca^2+^ stimulates glycolysis and Ca^2+^ uptake by activating CaMK and Cn. CaMK activates gene expression by CREB phosphorylation and by the activation of the AMPK-regulated pathway, whereas Cn activates NF-κB- and NFAT-dependent gene expression. Additionally, mtDNA depletion, mitochondrial chaperone inhibition, or mitochondrial protease inhibition induce the expression of mitochondrial chaperonins, known as UPR^MT^, in nematodes, although the molecular mechanism is not fully clarified in mammals. ATF4 is activated in response to mitochondrial ROS production via PERK and/or GCN2 to upregulate chaperone expression, amino acid synthesis and antioxidant synthesis. Increased cytoplasmic ROS activates Nrf2 and induces antioxidant gene expression. AMPK, AMP-activated protein kinase; ATF4, activating transcription factor 4; CaMK, calmodulin kinase; Cn, calcineurin; CREB, cAMP response element-binding protein; FOXO, forkhead box O; NFAT, nuclear factor of activated T-cells; NF-κB, nuclear factor-κB; PGC1α, PPARγ coactivator 1α; ROS, reactive oxygen species.

Mitochondrial retrograde signaling (MRS), which was originally described in yeast, encompasses a variety of mechanisms that signal the mitochondrial abnormalities to the nucleus and induce anterograde mechanisms (i.e., from the nucleus to the mitochondria mechanisms) that act in protection against these anomalies. In mammals, MRS includes the response to the mitochondrial unfolded protein (UPR^MT^),^(6,7)^ energetic stress,^(8)^ Ca^2+^ release^(9)^ and ROS production^(10)^ (Fig. 1). In this review, after first describing the general overview of the yeast and mammalian MRS, we will focus on the role of the ATF (activating transcription factor) 4 signaling pathway in the quality control and homeostasis of mitochondria in mammalian species (Fig. 1).

## Overview of MRS in Yeast

**Fig. 2:**
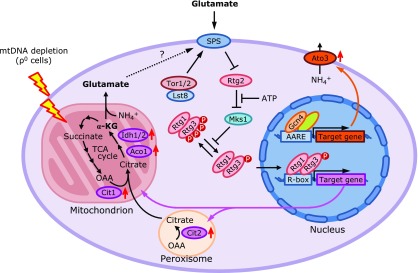
Activation of MRS reconfigures mitochondrial metabolism in yeast. Glutamate starvation and mitochondrial respiratory deficiency in mtDNA-depleted cells (ρ^0^ cells) activate MRS to express genes involved in mitochondrial TCA cycle (*CIT1*, *ACO1* and *IDH1/2*) and the peroxisomal glyoxylate cycle (*CIT2*). When the RTG pathway is not in operation, the basic helix-loop-helix leucine zipper transcription factors, Rtg1 and Rtg3 are sequestered to the cytoplasm where Rtg3 is hyperphosphorylated. Mks1 promotes Rtg3 hyperphosphorylation by somehow preventing the Rtg3 dephosphorylation and negatively regulates the pathway. When the retrograde pathway is turned on, Rtg3 becomes partially dephosphorylated, enters the nucleus together with Rtg1 and binds R box to activate transcription. Rtg2, a cytoplasmic protein with an N-terminal ATP-binding domain, localizes upstream of Rtg1 and Rtg3. In the absence of Rtg2, *CIT2* is no longer responsive to mitochondrial abnormality. Rtg2 binds to and inactivates Mks1, enabling for activation of Rtg1/3 and the RTG pathway. Physiological concentrations of ATP dissociates Mks1 from Rtg2 and negatively regulates the pathway.^(109)^ The SPS complex is a sensor for external amino acids and important for the glutamate repression of the RTG pathway and the inactivation of the SPS sensor system results in Rtg2-dependent increase of *CIT2* expression and a loss of glutamate repression. Lst8, which is an integral component of the target of rapamycin (TOR) complexes, is also a negative regulator of the RTG pathway and regulates the RTG pathway at two points: upstream and downstream of Rtg2. Rtg1/3-dependent gene expression is activated in cells in which TOR signaling mediated by the PI kinase-related kinases, Tor1 and Tor2, is inhibited by the immunosuppressant rapamycin. Mitochondrial dysfunction induces alternative retrograde signaling pathway to induce *ATO3* expression in a manner dependent on SPS and Gcn4 but not Rtg2. AAP, amino acid permease; ACO1, aconitate hydratase; α-KG, α-ketoglutarate; ATO3, ammonia transport outward 3; CIT, citrate synthase; IDH, isocitrate dehydrogenase; Lst8, lethal with sec thirteen 8; Mks1, multicopy kinase suppressor 1; OAA, oxaloacetate; RTG, retrograde regulation; SPS, Ssy1p-Ptr3p-Ssy5.

Historically, MRS has been extensively studied and characterized in yeast. Yeast can survive and proliferate without mitochondrial DNA (i.e., ρ^0^ cells) when cultured in the medium rich in the fermentable carbon sources such as glucose. In the respiration defective yeast cells such as ρ^0^ cells, MRS is used to readjust the need for carbon and nitrogen metabolism.^(11)^ In those cells, the flux of the TCA cycle decreases because the succinate dehydrogenase reaction cannot proceed in the absence of the intact mETC. In yeast, the TCA cycle intermediate, α-ketoglutarate is important for the intracellular synthesis of glutamate, which together with glutamine, are utilized as a precursor for nucleotides and other amino acids providing essentially all the nitrogen source for the biosynthetic needs.^(12)^ To replenish α-ketoglutarate in the cells, ρ^0^ cells induce an array of genes of an anaplerotic pathway, which includes the robustly induced peroxisomal glyoxylate cycle genes (Fig. 2).^(13)^ As such, the inhibition of MRS in ρ^0^ cells renders yeast glutamate auxotrophs. *CIT2*, a gene important for the peroxisomal glyoxylate cycle, and several other MRS target genes are controlled by Rtg1p, Rtg2p and Rtg3p^(13,14)^ (hereafter called RTG pathway). Rtg2p is indispensable for the activation of the bHLH-Zip transcription factors, Rtg1p and Rtg3p in ρ^0^ cells. Marked upregulation of the *CIT2* gene, a canonical target gene in the RTG pathway, in ρ^0^ cells can be reversed by the addition of glutamate in the medium.^(15)^ Thus, glutamate suppresses the RTG pathway in yeast, although the precise amino acids responsible for this glutamate repression of RTG target genes remains elusive, considering the extensive interconversion of the glutamate to glutamine and the related amino acids. SPS (*SSY1*, *PTR3*, *SSY5*) complex is an external amino acid sensor in the plasma membrane.^(16)^ SPS is required for the glutamate repression of the RTG pathway genes and *CIT2* gene is constitutively expressed in the SPS-deficient cells (Fig. 2). Knorre *et al.*^(17)^ discussed in the recent review how yeast specifically detects mitochondrial functional disturbances. As described above, it is reasonable to monitor the levels of glutamate as a signal for mitochondrial disturbance because glutamate as well as arginine is synthesized in the mitochondria. However, RTG pathway is also activated by inhibition of target of rapamycin (TOR) which is a main nutrient sensor in the cytoplasm. Therefore, the detection of altered amino acid levels by RTG pathway might not be specific to mitochondrial disturbance. Other mitochondrial products such as ATP and iron-sulfur cluster as well as mitochondrial derived peptides also can be utilized for the indicators of mitochondrial function.^(17)^ Actually, low ATP concentration dissociates Rtg2 from Mks1 and activates RTG pathway (Fig. 2). However, decrease of ATP is possible without mitochondrial disturbance cultured in the medium with high fermentable carbon sources. Thus, RTG response is not apparently specific to mitochondrial disturbance and influenced by different cellular contexts and metabolic status. In contrast, nematode ATFS-1 system directly monitors the disturbance of mitochondria by surveying the efficiency of mitochondrial protein import efficiency that depends on mitochondrial membrane potential^(18)^ (also discussed later). Homologous systems to ATFS-1 are not currently known in yeast or mammalian cells, and the same issues of specificity may also apply to the certain MRS pathways of mammals.

Other than RTG-dependent genes, *ATO3*, a gene important for ammonium excretion, is also upregulated in ρ^0^ cells. Gcn4, a master regulator of an amino acid starvation response in yeast, contributes to both basal and ρ^0^-inducible expression of *ATO3* and the SPS-dependent signal specifically contributes to the ρ^0^-inducible expression of *ATO3*.^(19)^ Gcn4 is also activated in yeast lacking the mitochondrial AAA protease gene *AFG3* and leads to the extension of longevity.^(20)^

## Overview of Mammalian Mitochondrial Retrograde Signaling

The roles of mitochondria and MRS could be different and diverse between species, especially between single cell eukaryotes and complex multicellular organisms such as mammals. In metazoans, mitochondria are essential not only for embryonic development but also for various functions in the adult organisms, including inflammation, immunity and apoptosis. In mammals, MRS has been often discussed in terms of quality control and adaptive mitochondrial biogenesis. For example, mitochondrial disturbance reduces ATP generation and activates AMPK, which activates PPARγ coactivator (PGC) 1α-mediated mitochondrial biogenesis to counteract the ATP decrease (Fig. 1). Mitochondria function as intracellular Ca^2+^ stores not only for buffering cytoplasmic Ca^2+^ in resting cells but also for stimulating ATP synthesis by activating Ca^2+^ transport and Ca^2+^-dependent enzymes in the TCA cycle and OxPhos in activated myocytes.^(21)^ Decreased mitochondrial respiration results in the loss of mitochondrial membrane potential (MMP), which reduces mitochondrial Ca^2+^ influx and leads to a sustained increase in cytoplasmic Ca^2+^ and the activation of calcineurin (Cn) and calcium/calmodulin-dependent kinase (CaMK). Cn activates glycolysis and OxPhos by expressing glucose transporter 4 (Glut4) and cytochrome c oxidase subunit 5B (COX5B) via NF-κB and NFAT, respectively.^(22,23)^ CaMK activates CREB, which induces the expression of cytochrome c, COX2, PGC1α and mitochondrial transcription factor A (TFAM) to increase OxPhos activity and mitochondrial biogenesis.^(24)^ CaMK also activates AMPK, which regulates PGC1α and FOXO, leading to the activation of mitochondrial biogenesis. Many environmental stresses, including UVA, induce the mitochondrial-derived ROS that activates the Nrf2 pathway.^(25)^ In addition, carbon monoxide administration reportedly activates cardiac Nrf2 by the generation of mitochondrial ROS.^(26)^

## ATF4 Act as a Stress-responsive Transcription Factor

Activating transcription factor 4 (ATF4) is a basic leucine zipper (bZip) transcription factor belonging to the ATF/CREB subfamily. ATF4 is activated by various stresses, such as endoplasmic reticulum (ER) stress and amino acid deficiency.^(27)^ ATF4 forms homodimers or heterodimers with other transcription factors, such as C/EBPβ, and functions as a transactivator or repressor. The ATF4/C/EBPβ heterodimer recognizes the consensus DNA sequence [amino acid responsive element (AARE) or C/EBP-ATF response element (CARE)], whose consensus sequence is TGATGNAAN. ATF4/C/EBPβ heterodimer activates the expression of downstream target genes, such as asparagine synthetase (*ASNS*) and tribbles homolog 3 (*Trib3*). ATF4 modulates many important biological processes, such as antioxidant response, apoptosis, energy homeostasis, autophagy and osteogenesis. Several abnormalities, including osteoporosis, microphthalmia and abnormal hematopoiesis, found in ATF4 knockout (KO) mice reflect the significant role of ATF4 in mammalian development. ATF4 activation by stress signals is mediated by a conserved signaling pathway called the integrated stress response (ISR). Ser51 phosphorylation of eukaryotic initiation factor (eIF) 2α by various eIF2α kinases leads to the selective translation of ATF4 and global translation inhibition.^(28)^ In mammalian cells, four stress responsive eIF2α kinases, heme-regulated inhibitor of translation (HRI), dsRNA-activated protein kinase (PKR), PKR-like endoplasmic reticulum kinase (PERK) and general control nonderepressible-2 (GCN2), catalyze eIF2α phosphorylation in response to respective stress signals. Each eIF2α kinase is activated by different stress signals, for example, HRI (heme deficiency), PKR (viral infection), PERK (ER stress), and GCN2 (amino acid deficiency). Notably, eIF2α phosphorylation decreases GTP bound eIF2 (GTP-eIF2) by sequestering eIF2α. When GTP-eIF2 is abundant, global translation is sustained, while ATF4 translation is inhibited due to two upstream open reading frames (uORFs) in the 5'-untranslated region (5'-UTR) of ATF4 mRNA. In contrast, global translation is slowed down when the GTP-eIF2 level is low, but low GTP-eIF2 allows the ribosome to bypass the inhibitory uORF and upregulate selective ATF4 translation.

## Activation of ATF4 in Mitochondrial Diseases

Mitochondrial dysfunction caused by mutations in mitochondrial DNA (mtDNA) is related to a variety of diseases, including Mitochondrial myopathy, Encephalopathy, Lactic Acidosis, Stroke-like episodes (MELAS) and Neurogenic Ataxia and Retinitis Pigmentosa (NARP) syndromes.^(2)^ To recapitulate the cellular response to mitochondrial gene mutations, Fujita *et al.*^(29)^ generated human hybrid 143B osteosarcoma cells harboring mitochondria with the MELAS type mutation (3243A>G mutation within the mitochondrial tRNA gene for leucine), called 2SD cells, and the NARP type mutation (8993T>G mutation resulting in an amino acid change from a highly conserved leucine at position 156 to arginine in subunit 6 of mitochondrial F_o_F_1_-ATPase), called NARP3-1 cells. The authors showed that ATF4 is activated in these cells, accompanied by the induction of its target protein CHOP and ASNS.^(29)^ These authors also showed that the induction of CHOP and ASNS is also observed by rotenone, an inhibitor of complex I of mETC. Cortopassi *et al.*^(30)^ systemically examined the cellular response to mitochondrial mutations by using at least 2 representative cell types of five mitochondrial diseases, including Leber’s Hereditary Optic Neuropathy (LHON), Friedreich’s ataxia (FRDA), NARP, Myoclonic Epilepsy with Ragged Red Fibers (MERRF), and Kearns–Sayre Syndrome (KSS). The top-ranked inducible gene category was related to proliferation and cell cycle, as well as the unfolded stress response, which is regulated by ATF4.

## Activation of ATF4 by Mitochondrial Functional Disturbance

**Fig. 3:**
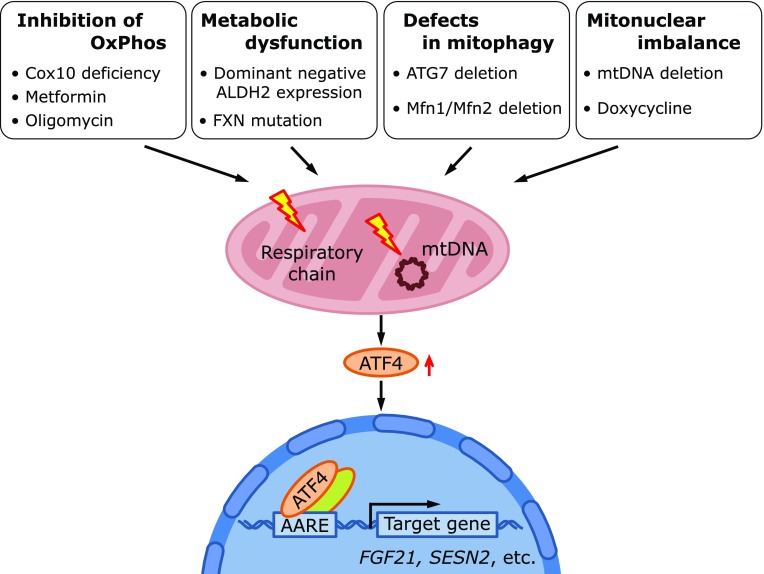
ATF4 is activated by the perturbation of mitochondrial function. Perturbation of mitochondrial function induces ATF4 activation. Genetic alteration or inhibition of OxPhos enzymes increases ROS production, which may induce ATF4 activation. ATF4 is also activated by the dysregulation of other metabolic pathways, such as reactive aldehyde accumulation by dominant negative ALDH2 expression or perturbed iron homeostasis by FXN deficiency. Mitophagy regulates mitochondrial quality control by eliminating damaged, depolarized mitochondria. Defects in mitophagy induce the accumulation of impaired mitochondria and ATF4 activation. An imbalance in mitochondrial- and nuclear-encoded mitochondrial proteins caused by mtDNA deletion or mitochondrial ribosome inhibition by doxycycline confers ATF4 activation. FXN, frataxin; OxPhos, oxidative phosphorylation.

Accumulating evidence has demonstrated that several distinct stimuli that disturb mitochondrial functions activate the ATF4 pathway (Fig. 3 and Table 1).

### Activation of ATF4 by the inhibition of OxPhos

 Familial progressive external ophthalmoplegia (PEO) is a progressive adult-onset mETC deficiency with multiple mtDNA deletions.^(31,32)^ Deletor mice are models of PEO in which the dominant mutation of the mtDNA helicase Twinkle leads to the deletion of mtDNA and the late onset deficiency of the mETC.^(33)^ In deletor mice, as well as cytochrome c assembly factor 10 (Cox10)-deficient mice, ATF4 target genes, including fibroblast growth factor (FGF) 21 and Mthfd2, are induced in the muscle.^(34)^ AKT1 phosphorylation of both threonine 308 and serine 473 is activated in the skeletal muscle of deletor mice, indicating the involvement of AKT1 in mitochondrial stress-induced ATF4 activation. These authors further showed that the overexpression of PGC1α ameliorates the mETC deficiency and decreases the induction of FGF21 and Mthfd2 in Cox10-deficient mice. Metformin, a glucose-reducing agent clinically used to treat diabetes, inhibits mETC. Kim *et al.*^(35)^ showed that metformin induced FGF21 gene expression and ATF4 protein levels in the mouse liver. Metformin inhibits complex I activity in rat hepatoma FaO cells and induces FGF21 gene expression via ATF4 activation in FaO cells and mouse embryonic fibroblasts (MEFs).^(35)^ Furthermore, mitochondrial antioxidant Mito-TEMPO, with superoxide and alkyl radical scavenging activity, inhibits metformin-induced ATF4 activation in FaO cells, suggesting the role of mitochondrial-derived ROS. Furthermore, metformin-induced ATF4 activation is PERK-dependent, indicating that metformin induces ATF4 in the mitochondrial ROS-PERK pathway (discussed later). Overexpression of uncoupling protein 1 (UCP1) in skeletal muscle improves insulin resistance and prolongs the lifespan of mice fed a high-fat diet.^(36)^ Furthermore, Keipert *et al.*^(37)^ demonstrated that UCP1 overexpression in the skeletal muscle activates ATF4 and FGF21 gene expression. These authors showed that both UCP1 overexpression and the inhibition of mitochondrial respiration, both potentially decrease mitochondrial membrane potential, activate ATF4 in C2C12 myotubes. LHON is caused by the mutation of complex I subunits. Mimicking LHON, Silva *et al.*^(38)^ demonstrated that the complex I inhibitor, rotenone induces ATF4, which is associated with PERK activation in human oligodendrocytes. Rotenone also induced PERK-dependent JNK activation in these cells. In HCT116 cells, the inhibition of ATP synthase by oligomycin, that causes mitochondrial hyperpolarization in contrast to the mETC inhibitor, induces the reprogramming of energetic metabolism, where mitochondrial functions are suppressed.^(39)^ Oligomycin-induced AMPK and ATF4 may be responsible for mitochondrial repression. The inhibition of the mETC complex I and III induces SESN2 expression in cancer cell lines in an ATF4-dependent manner.^(40)^

### Activation of ATF4 by metabolic dysfunction

The pioneering work of Endo *et al.*^(41)^ demonstrated that the accumulation of reactive aldehydes due to the expression of a dominant negative type of mitochondrial aldehyde dehydrogenase 2 (ALDH2) activates ATF4 in the heart and provokes the cytoprotective response against the ischemic reperfusion injury of the heart. FRDA is caused by mutations in the FXN gene that encode the mitochondrial matrix protein frataxin, which is indispensable for iron-sulfur cluster biosynthesis in the mitochondria. In FXN muscle-specific knockout mice, ATF4 is activated by still unknown mechanisms preceding the functional and anatomical deterioration of the heart.^(42)^

### Activation of ATF4 by defects in mitophagy

 Mitophagy is an important quality control system for damaged or functionally deficient mitochondria. In the muscle-specific KO mice of ATG7, mitochondrial function, assessed by state 3 and state 4 respiration, was decreased, and FGF21 gene expression was activated via ATF4.^(43)^ Parkin and PINK1 are mutated in familial types of Parkinson’s disease and are involved in the quality control of mitochondria. In *Drosophila* ATF4 is induced in the heads of pink1 and parkin mutants, which are well-known models of Parkinson’s disease in *Drosophila* and are defective in mitochondrial function.^(44)^

### Activation of ATF4 by defects in mitochondrial dynamics

Mitochondrial dynamics of fusion and fission are intimately involved in mitochondrial function. Furthermore, the specific deletion of Mitofusin 1 (Mfn1) and 2 (Mfn2) also activates ATF4 signaling in the muscle.^(43)^ This effect is most likely mediated by the activation of Perk because the knockout of Mfn2 causes ER stress and activates Perk.^(45)^ Consistently, Optic Atrophy 1 (OPA1) knockout also induces ER stress and FGF21.^(46)^

### Activation of ATF4 by mito-nuclear imbalance

 Mitonuclear protein imbalance can be elicited by various mechanisms, including mtDNA mutation, mitochondrial translational inhibition or nuclear gene repression, and may contribute to the longevity of many species.^(47)^ The mitochondrial translation inhibitor doxycycline (DOX) and other tetracyclines also cause ATF4 activation in various cancer cells.^(48,49)^ Bao *et al.*^(50)^ demonstrated that mitochondrial DNA depletion, induced by the expression of a dominant negative DNA polymerase γ mutant or ethidium bromide, activates ATF4 in HEK293 cells.

## Mammalian UPR^MT^ and ATF4

UPR occurs when the amount of misfolded proteins exceeds the protein folding capacity of the cell. Originally, UPR was described with respect to the ER. However, the UPR of mitochondria has recently been reported.^(6)^ This pathway was first described in mammals, in which mitochondrial chaperones are induced by mitochondrial DNA deletion,^(51)^ but thereafter, most mechanisms have been clarified in *C. elegans*. Mitochondria possess their own chaperones and proteases and their gene expression is activated by UPR^MT^. In *C. elegans*, unfolded proteins are degraded by the mitochondrial protease Clp, and the resulting peptides are transported by the inner mitochondrial peptide transporter HAF-1 and subsequently diffused to the cytoplasm. The transcription factor ATFS-1 typically translocates to the mitochondria and is degraded by LON protease, but the decreased mitochondrial import of ATFS-1 leads to nuclear translocation of ATFS-1 and activates UPR^MT^. Both the reduced mitochondrial protein import and ATFS-1 nuclear translocation rely on HAF-1 and peptides efflux; however, the precise mechanism and tight regulation remain unclear. Although ATFS-1 and HAF-1 do not exist in mammals, emerging evidence has shown that a functionally homologous system also exists in mammals. Munch *et al.*^(52)^ showed that the inhibitors of mitochondrial chaperonin, HSP90/TRAP1 GTPP and LON protease CDDO, lead to global changes in gene expression that are associated with defects in mitochondrial pre-RNA processing leading to the inhibition of mitochondrial protein synthesis. The genes upregulated by acute mammalian UPR^MT^ stimuli include mitochondrial chaperonins HSPD1 and HSPE1, as well as several ATF4 target genes, such as CHOP. The promoters of genes induced by both GTPP and CDDO showed enrichment for binding sites for CHOP and ATF4, as well as mitochondrial UPR response element (MURE1 and MURE2), supporting the role of ATF4 in the mammalian UPR^MT^. ATF4 may be activated by the mito-nuclear imbalance caused by mitochondrial translational inhibition. However, these authors showed that the knockdown of any of four eIF2α kinases does not affect CHOP gene induction by GTPP, indicating an eIF2α phosphorylation-independent mechanism of ATF4 activation by GTPP.

## EIF2α Kinases and ROS as Candidate Upstream Regulators of ATF4 Activation by Mitochondrial Perturbation

Bao *et al.*^(50)^ showed that OxPhos inhibitors, such as antimycin and oligomycin, but not the mitochondrial protonophore CCCP, strongly activate ATF4 in HEK293 cells, indicating that OxPhos inhibitor-induced ATF4 activation involves redox stress but not ATP depletion and loss of MMP. Bouman *et al.*^(53)^ showed that mitochondrial dysfunction triggered by CCCP induces ER stress and activates ATF4-dependent Parkin gene induction via the UPR^ER^-PERK pathway in SH-SY5Y cells.^(53)^ Interestingly, Parkin is also induced by tunicamycin or thapsigargin and prevents the decrease of ATP synthase and cell viability by preventing mitochondria fragmentation. Therefore, Parkin is protective against ER-stress induced mitochondrial perturbation. Furthermore, metformin induces ATF4 in an AMPK-independent but PERK-dependent manner.^(35)^ It is increasingly clear that the mitochondria-associated membrane (MAM) plays a pivotal role in cellular signaling and communication between mitochondria and ER.^(54)^ Interestingly, CO activates ATF4 via PERK in a mitochondrial ROS-dependent manner, indicating that mitochondrial disturbance induces ATF4 via MAM communication.^(55)^ However, several data suggest the involvement of GCN2 as an upstream regulator of ATF4 activation, as reported in nematodes.^(56)^ Martinez-Reyes *et al.*^(39)^ found that GCN2 is activated by oligomycin and postulated that the GCN2-ATF4 pathway, as well as AMPK, is responsible for the metabolic adaptation (i.e., mitochondrial repression) to oligomycin in HCT116 colon cancer cells.^(39)^ Increased eIF2α phosphorylation by DOX was inhibited by GCN2 knockdown in HeLa cells.^(48)^ Wang *et al.*^(57)^ observed that mitochondrial dysfunction in gastric cancer cells enhanced cisplatin resistance by induction of the cysteine transporter xCT.^(57)^ These authors showed that the mitochondrial dysfunction-induced ATF4 activation is dependent on GCN2, but not PERK. Therefore, certain mitochondrial toxic stimuli, such as CCCP and complex I inhibitor, likely cause ER stress and induce ATF4 in a PERK-dependent manner, while other toxic stimuli against mitochondria induce ATF4 in GCN2- and cell-type dependent manners.

In a glucose deficient state, the anaplerotic pathway from pyruvate is decreased, resulting in the decrease of TCA cycle metabolites. Then, glutamate is used to provide α-ketoglutarate to replenish the TCA cycle (i.e., anaplerotic pathway) resulting in glutamate insufficiency.^(58)^ Indeed, the GCN2-ATF4 pathway is induced by glucose deficiency, associated with the decreased intermediates of the TCA cycle and several amino acids.^(59)^ In proliferating cells, the inhibition of ETC results in aspartate deficiency due to oxaloacetate insufficiency.^(60)^ Therefore, as in yeast amino acid starvation can also be a signal for MRS in mammals, although it is heavily dependent on the availability of the amino acids from the environment. This hypothesis should be examined in future analyses. Considering that the nematode GCN-2-ATF4 pathway is also activated in response to mitochondrial perturbation,^(56)^ the GCN-2-ATF4 pathway may be an evolutionarily conserved pathway that responds to mitochondrial perturbation by sensing the activity of the TCA cycle.

## Mechanisms of ATF4-mediated Mitochondrial Quality Control

ATF4 regulates genes related to intracellular amino acid homeostasis, such as amino acid transporter and amino acid synthase, as well as antioxidant genes, such as xCT.^(27)^ Furthermore, ATF4 regulates apoptosis-related genes, such as CHOP, and enhances apoptosis when ER stress is severe or prolonged.^(61)^ However, how ATF4 regulates mitochondrial function is only partly understood. If ATF4 is activated by mitochondrial stimuli and protective against mitochondrial dysfunction, then what are the underlying mechanisms? Several genes important for mitochondrial functions are regulated by ATF4.

### Parkin

The PINK1-Parkin pathway plays an important role in mitochondrial quality control by sensing mitochondrial damage and subsequently inducing the mitophagy of the damaged mitochondria.^(62)^ As mentioned above, Bouman *et al.*^(53)^ showed that ATF4 regulates Parkin to promote mitochondrial quality control. These authors also showed that ATF4 binds to the CREB/ATF4 sites of the Parkin gene and that c-Jun negatively regulates Parkin gene expression in the same region.

### Mitochondrial folate-mediated one carbon unit (1C) metabolism, serine synthesis and transsulfuration pathway

**Fig. 4:**
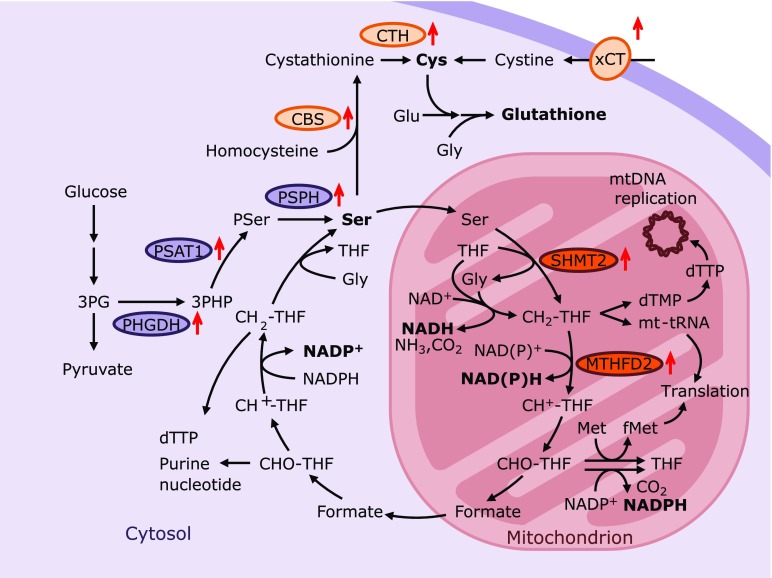
ATF4 regulates the serine synthetic and mitochondrial Folate-mediated 1C metabolism pathway. Several ATF4 target genes are involved in serine synthesis, 1C unit metabolism, purine synthesis and glutathione synthesis. The expression of PHGDH, PSAT1 and PSPH increases serine synthesis. Serine is incorporated into glutathione synthesis as well as into 1C unit metabolism. Expression of xCT, CTH and CBS increases glutathione synthesis. The expression of SHMT2 and MTHFD2 accelerates NADPH production, fMet synthesis and purine synthesis, which are important for mitochondrial redox homeostasis, mitochondrial translation and DNA replication, respectively. SHMT2-mediated tRNA methylation is important for the translation of mitochondria-encoded electrophile transport chain. In proliferating cells, CHO-THF and CH2-THF generated in the cytosol is utilized for purine and thymidine synthesis, respectively. 3PG, 3-phospho-glycerate; 3PHP, 3-phospho-hydroxypyruvate; CBS, cystathionine-β-synthase; CH_2_-THF, 5,10-methylene-THF; CH^+^-THF, 5,10-methenyl-THF; CHO-THF, 10-formyl-THF; CTH, cystathionase; dTMP, thymidine monophosphate; dTTP, thymidine triphosphate; fMet, *N*-formylmethionine; MTHFD2, methylene-tetrahydrofolate dehydrogenase 2; PHGDH, phosphoglycerate dehydrogenase; PSAT1, phosphoserine aminotransferase 1; PSer, 3-phospho-serine; PSPH, phosphoserine phosphatase; SHMT2, mitochondrial serine hydroxymethyl transferase 2; THF, tetrahydrofolate.

Folate-mediated 1C metabolism, and the serine synthesis and transsulfuration pathway, as well as the methylation pathway, are highly inter-related metabolic pathways.^(63)^ A recent analysis demonstrated that a majority of serine is metabolized in mitochondria, and mitochondrial formate production is critical for the cytosolic production of purines and thymidines in most cancer cell lines.^(64)^ ATF4 has recently been recognized as a central player regulating mitochondrial 1C metabolism gene expression, as well as the genes of the serine synthesis and transsulfuration pathway, as shown by the abovementioned results (Fig. 4).^(41,65)^ Considering the degenerative diseases in the brain, the *de novo* synthesis of serine (i.e., phosphorylated serine pathway) is important because the blood-brain barrier does not sufficiently transport dietary serine.^(66,67)^ In mitochondrial disease model mice, these pathways are upregulated, resulting in the remodeling of one-carbon metabolism.^(68)^ Additionally, ATF4 regulates these pathways to contribute to cancer progression by promoting purine biosynthesis.^(69)^ Importantly, mitochondrial 1C metabolism is also important for the mitochondrial synthesis of NADPH byproducts, essential cofactors for antioxidant and lipid biosynthesis in mitochondria.^(70,71)^ In the dominant negative ALDH2-expressing mice described above, the gene expression of Mthfd2 and Phgdh, which are rate-limiting enzymes of mitochondrial 1C metabolism and the serine synthesis pathway, respectively, is markedly induced.^(41)^ These authors observed the increase of cellular GSH, although mitochondrial NADPH generation is likely also increased, contributing to the oxidative stress-resistance phenotype of the mice. In parkin and pink1 mutant flies, the expression of Shmt2, Mthfd2, GCS P and T protein genes, which are involved in mitochondrial folate-mediated 1C metabolism generating mitochondrial NADPH production, is upregulated via ATF4 activation.^(44)^ Apparently, the activation of 1C metabolism does not lead to cell proliferation in this case but likely activates NADPH production in mitochondria. Furthermore, Shmt2 is important for the *de novo* synthesis of dTTP and mitochondrial DNA replication,^(72)^ as well as the translation of mitochondria-encoded ETC through the regulation of tRNA methylation, as recently reported in mammals.^(73)^ Moreover, ATF4 activation in the heart by Calcineurin catalytic subunit β1 (CnAβ1) overexpression (discussed later) protects mitochondrial protein oxidation and exerts protective function against pressure-overload mediated cardiac hypertrophy in a PHGDH and glutathione-dependent manner.^(74)^ Therefore, ATF4-regulated mitochondrial 1C metabolism may be important for mitochondrial biogenesis and mitochondrial antioxidant function (Fig. 4).

### FGF21

ATF4 induced the myokine FGF21, as shown by the abovementioned results.^(34,43)^ FGF21 has glucose-lowering and anti-lipidemic effects in obese animals, and this protein may have a good influence on metabolic syndrome and longevity. FGF21 is regulated by Akt in the skeletal muscle and is considered a stress hormone.^(75)^ Lehtonen *et al.*^(76)^ argue that FGF21 is massively induced by the defect of mitochondrial translation or the deletion of mtDNA, but not so strikingly by the defect of mETC. This is somewhat consistent with the report by Dogan *et al.*^(77)^ that a defect of mitochondrial aspartyl-tRNA causes functional abnormality of mETC in both the heart and skeletal muscle, but that FGF21 induction is observed only in the heart, arguing that FGF21 induction is not caused by the deficiency of mETC, but proteostasis abnormality in the mitochondria. Thus, how mitochondrial functional defects induces FGF21 gene expression will still remain to be clarified in the future. The effector mechanism of FGF21 is largely unknown, but this protein improves mitochondrial function via the AMPK-SIRT1-PGC1α pathway.^(78)^

### Others

ATF4 confers pro-survival signals under oxidative stress by regulating the gene expression of MKP-1 and inhibiting pro-apoptotic MAPK signaling.^(79)^

## Activation of the ATF4 Pathway by Extracellular Growth Signals

**Fig. 5:**
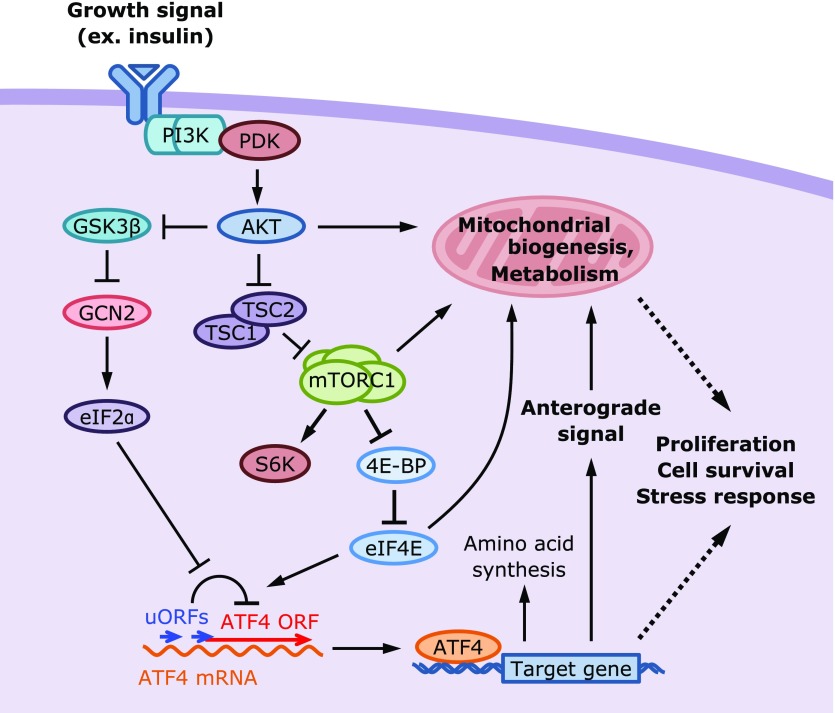
Growth signal activates ATF4 and mitochondrial metabolism. ATF4 activation is induced not only by stress signals that induce eIF2α phosphorylation and translational inhibition but also by growth signals that activate mTORC1 and accelerate translation. Growth signals activate AKT, which phosphorylates a number of substrates in cytoplasm and mitochondria. Phosphorylation of TSC1/2 activates mTORC1, which in turn phosphorylates S6K and 4E-BP to accelerate translation. Notably, mTORC1 activation induces ATF4 expression without eIF2α phosphorylation but likely via the depletion of translation re-initiation factors. ATF4 induces the gene expression in amino acid synthesis/transport and aminoacyl-tRNA synthesis in response to the demand for protein synthesis. 4E-BP, eukaryotic initiation factor 4E binding protein; TSC, tuberous sclerosis.

Recently, it is increasingly clear that growth signals activate the ATF4 pathway, especially in cancer cells (Fig. 5). Considering that the ATF4 pathway regulates amino acid-starvation responses, including the induction of the cell surface amino acid transporters important for cellular nutrition and growth, it is reasonable that extrinsic growth signals also utilize the ATF4-mediated pathway for cell growth.^(80)^ In the hippocampal cell line HT22, the PI3K inhibitor LY294002 reduced eIF2α phosphorylation, which was associated with decreased expression of xCT and ATF4. Furthermore, the GSK3β inhibitor, lithium chloride rescues the PI3K inhibition-induced ATF4 decrease in a GCN2-dependent manner, indicating that the PI3K-Akt-GSK3β-GCN2-eIF2α pathway is involved in the basal expression of ATF4 (Fig. 5). Consistent with these observations, insulin robustly activates ATF4 in HT22^(81)^ and moderately activates ATF4 in MEFs.^(69)^ Interestingly, mTORC1, another target of Akt, activates ATF4 to subsequently activate purine synthesis in cancer cells.^(69)^ Furthermore, the neuron-specific deletion of TSC2, a negative inhibitor of mTORC1, activates mTORC1 signaling and increases both ATF4 and MTHFD2 gene expression in the brain.^(69)^ The induction of ATF4 by tunicamycin was abolished in Ser51Ala eIF2α mutant knock-in MEFs [eIF2α (A/A) MEFs]. However, the insulin-induced ATF4 accumulation was inhibited by the mTORC1 inhibitor rapamycin but only partially diminished in eIF2α (A/A) MEFs compared to wild-type MEFs, indicating a non-canonical mechanism for mTOR-mediated ATF4 activation.^(69)^ Recently, Park *et al.*^(82)^ also reported that mTORC1 activates ATF4 by regulating eIF4E-binding protein (4E-BP). Furthermore, Akt overexpression induces FGF21 expression, suggesting that Akt somehow activates ATF4 *in vivo*.^(83)^ Notably, mTORC1 regulates mitochondrial respiration,^(84)^ and Akt is localized to mitochondria after PI3K stimulation,^(85)^ suggesting the potential involvement of mitochondria in these reactions (Fig. 5). Interestingly, a recent study revealed that ATF4 activation is executed by mTOR activation in the affected muscle fibers in mitochondrial myopathy, suggesting that mTOR is localized upstream in ATF4 activation in response not only to extrinsic growth signals but also to mitochondrial disturbance.^(86)^ However, CnAβ1, a constitutively active form of calcineurin catalytic subunit lacking the C-terminal autoinhibitory domain, also activates ATF4 via the mTORC2-AKT-mTORC1 pathway in the heart, and the overexpression of CnAβ1 improves cardiac function after cardiac infarction^(87)^ or pressure overload-mediated cardiac hypertrophy.^(74)^ Interestingly, CnAβ1 is induced by IGF-1 in the cardiac heart.^(88)^

## Mechanism of AKT Activation by Mitochondrial Stress

As described above, mitochondrial stress activates AKT, but its mechanisms are not fully understood. Mitochondrial depletion or CCCP induced the expression of both insulin and IGF-1 receptor (IGF-1R) but selectively activated IGF-1R in a Cn-dependent manner in the myoblast cell line C2C12, which is responsible for Akt activation leading to enhanced glucose uptake and tumor invasion.^(89)^ Intriguingly, this induction of IGF-1R by mitochondrial stress has been observed in many cancer cell lines, including A549, H9C2 cardiomyocytes, and NIH3T3 cells. Murata *et al.*^(90)^ reported that PINK1 overexpression induces the phosphorylation of AKT serine 473 via mTORC2, suggesting that mitochondrial stress activates AKT via activation of PINK1. However, the pioneering work of Kazemi *et al.*^(91)^ demonstrated that the activation of eIF2α phosphorylation leads to the activation of PI3K. This effect may be related the inhibition of the negative feedback regulation of PI3K via mTORC1. They demonstrated that eIF2α phosphorylation suppresses mTORC1 and derepressed PI3K activates mTORC2-dependent AKT phosphorylation in an ATF4-independent manner.^(92)^ Thus, interferons and thapsigargin induce S6 phosphorylation in a partly PKR- and PERK-dependent manner, respectively, in MEFs.^(91)^ Therefore, eIF2α phosphorylation might somehow activate PI3K in response to mitochondrial disturbances.

## ATF4 Activation as the Earliest Change during Degenerative Diseases and Toxic Stimuli - a Prosurvival Role of the ISR-ATF4 Pathway

Prolonged ATF4 activation often leads to apoptosis and is proposed to accelerate the degenerative diseases,^(61,93)^ but several reports have indicated that the primary function of ATF4, at least in the early phase of stress, is cytoprotective. As noted above, Huang *et al.*^(42)^ observed that early ATF4 activation precedes the onset of heart remodeling by using muscle-specific FXN KO mice, indicating that ATF4 acts as a cytoprotective factor. Krug *et al.*^(65)^ performed combined metabolomics and transcriptome analysis in human postmitotic dopaminergic neurons and showed that ATF4 is activated in the early phase (i.e., 24 h) after the treatment of mitochondrial toxicant MPP^+^ well before the onset of the sign of the toxicity was observed (i.e., after 48 h). Evstafieva *et al.*^(94)^ examined the role of the ISR-ATF4 pathway in the protection against apoptosis in cancer cells. Mitochondrial complex III inhibitor myxothiazol activates the ATF4 pathway in HCT116 cells from the early phase of the time course (~5 h). The prolonged inhibition of complex III, which specifically inhibits *de novo* pyrimidine synthesis, activates the p53-mediated inhibition of ATF4 gene expression, leading to cell death by apoptosis. The authors argued that ATF4 acts as a protective factor against mitochondrial inhibitor-induced apoptosis. In summary, these results showed that ATF4 is activated by mitochondrial stress at the early, pre-symptomatic stage of disease progression and is protective against the ensuing damage. We propose that ATF4 activation is a biomarker for mitochondrial stress that shows promise for intervention.

## Prevention of Age-related or Mitochondrial Diseases by Nrf2 Activation

### Role of Nrf2 in mitochondrial quality control

**Fig. 6:**
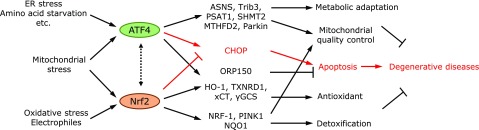
Cooperative gene regulation for mitochondrial quality control by Nrf2 and ATF4. It is becoming clear that mitochondrial stress activates ATF4. In certain tissues, such as in the heart, mitochondrial stresses can also activate Nrf2 in a tissue-dependent manner. ATF4 and Nrf2 induce certain stress response genes (ER chaperone and amino acid synthetic genes by ATF4 and antioxidant genes by Nrf2), but both factors regulate the genes that are important for the mitochondrial quality control. Notably, Nrf2 and ATF4 physically interact. Nrf2 inhibits ATF4 binding to the CHOP promoter region and cooperatively regulates antioxidant genes, such as HO-1 and xCT, and the anti-apoptotic gene ORP150.

Nrf2 regulates the expression of hundreds of cytoprotective genes to counteract endogenously or exogenously generated oxidative stress.^(95)^ These gene products mainly diminish oxidative stress in the cytosol, regulating the cytosolic amount of glutathione and NADPH. However, recent studies are beginning to uncover the important roles of Nrf2 in mitochondrial quality control and biogenesis by regulating the expression of NRF-1, PINK1 and TFAM (Fig. 6).^(96–99)^ The effect of Nrf2 in mitochondrial quality control and biogenesis is particularly evident in the stressed conditions, such as under oxidative stress or bacterial infection, as no activation of the abovementioned genes in the normal homeostatic conditions, such as in Keap1 knockout cells. Nrf2 is also specifically activated in the heart of Complex IV-deficient *surf1*^−/−^ mice suggesting that Nrf2 plays a role in mitochondrial retrograde responses in a tissue-specific manner.^(100)^

### Utilization of crosstalk between Nrf2 and ATF4 in the prevention of the degenerative diseases

We have previously identified ATF4 as a Nrf2-interacting factor in a yeast two-hybrid system by using Nrf2-C-terminal region (including Neh6, Neh1 and Neh3) as bait (data not shown). He *et al.*^(101)^ demonstrated that Nrf2 and ATF4 cooperatively induce heme oxygenase 1 (HO-1) expression, showing that Nrf2 and ATF4 bind to Stress Response Element in the HO-1 enhancer, although with a lower binding affinity compared to that of the Nrf2-small Maf heterodimer in the EMSA assay by using recombinant proteins. We also tested the binding of the Nrf2-ATF4 heterodimer to the antioxidant response element in the EMSA assay by using recombinant proteins, but we did not detect any significant binding. Instead, we showed that Nrf2 and ATF4 cooperatively activate the gene expression of xCT, a subunit of the xc^−^ cysteine transporter, in bladder cancer cell lines. Nrf2 and ATF4 independently bind to Nrf2 (ARE) and ATF4 (AARE) binding sites in the regulatory region and cooperatively regulate xCT gene transcription through their physical interaction.^(102)^ Furthermore, Nrf2 and ATF4 cooperatively induce other antioxidant enzymes, such as γ-glutamyl cysteine synthase subunits and glutathione reductase, in response to the antioxidant carnosic acid (Mimura J *et al.*, 2018, unpublished observation). Zong *et al.*^(103)^ examined the sensitivity of thyroid cancer cells to proteasome inhibitors and showed that Nrf2 inhibits apoptosis by negatively affecting the positive effect of ATF4 in CHOP expression.^(103)^ These authors demonstrated that Nrf2 affects ATF4 binding to the CHOP regulatory regions. Furthermore, Nrf2 and ATF4 cooperatively regulate the expression of the anti-apoptotic molecular chaperone ORP150, localized to both the ER and mitochondria.^(104)^ Thus, these results demonstrated that Nrf2 cooperatively induces antioxidant enzymes and anti-apoptotic genes with ATF4 while inhibiting the adverse effects of ATF4, suggesting the preventive effect and the intervention timing of Nrf2-mediated therapies by using Nrf2 activating phytochemicals, such as sulforaphane, or drugs, such as dimethyl fumarate (Fig. 6). Furthermore, ATF4 activation may result in the net transfer of cytosolic NADPH into the mitochondria in post-mitotic cells, such as in the heart and neurons, resulting in increased NADP^+^/NADPH in the cytosol (Fig. 4). The concomitant activation of Nrf2 catalyzed the reaction of cytosolic NADP^+^ to NADPH by regulating the pentose phosphate pathway and malic enzymes.^(95)^ In addition to the abovementioned mechanisms, Nrf2 also regulates ATF4 gene expression in cancer cells and indirectly regulates the serine synthetic pathway in cancer cells, leading to poor prognosis in non-small cell lung cancer patients.^(105)^ Nrf2 may also regulate MTHFD2, as Nrf2 knockdown downregulates MTHFD2 gene expression in A549 lung cancer cells.^(106)^ However, the physiological roles of the mechanisms still need further verification in the future.

## Concluding Remarks

In this review article, we discussed the role and mechanism of ATF4 activation during disease progression and environmental stresses, as well as its role downstream of extrinsic growth signals. We propose that the ISR-ATF4 pathway represents a pro-survival response against mitochondrial stress, and ATF4 is responsible for homeostatic and mitohormesis responses in mammals.

## Figures and Tables

**Table 1:** *In vivo* evidence of ATF4 activation by mitochondrial stress

	Tissue	Evidence	Ref	Others
Transgenic mice systemically expressing dominant patient mutation of mitochondrial DNA helicase (deletor mice)	Muscle	Mthfd2 and Fgf21 gene induction	(29)	Associates with AKT activation
Cox10 deficient mice	Muscle	Mthfd2 and Fgf21 gene induction	(29)	Associates with AKT activation
Metformin treatment	Liver	Increased ATF4 protein and Fgf21 gene induction	(30)	
Skeletal muscle-specific UCP1 overexpression	Skeletal muscle	Increased ATF4 protein and Fgf21 gene induction	(32)	
Systemic expression of dominant negative ALDH2	Heart	Increased ATF4 target gene induction	(36)	Heterozygous deletion of ATF4 diminished the response
Frataxin knockout	Muscle	Increased ATF4 target gene induction	(37)	
Skeletal muscle-specific ATG7 knockout	Skeletal muscle	Increased Fgf21 gene induction in skeletal muscle	(38)	
Skeletal muscle-specific Mfn1 and Mfn2 double knockout	Skeletal muscle	Increased ATF4 protein and eIF2α phosphorylation in skeletal muscle	(38)	

## Abbreviations

AARE: amino acid responsive element
ATF4: activating transcription factor 4
ASNS: asparagine synthetase
CaMK: calcium/calmodulin-dependent kinase
CARE: C/EBP-ATF response element
Cn: calcineurin
CnAβ1: calcineurin catalytic subunit β1
Cox10: cytochrome c assembly factor 10
COX5B: cytochrome c oxidase subunit 5B
4E-BP: eIF4E-binding protein
eIF2α: eukaryotic initiation factor 2α
FGF: fibroblast growth factor
FRDA: Friedreich’s ataxia
GCN2: general control nonderepressible-2
Glut4: glucose transporter 4
HRI: heme-regulated inhibitor of translation
ISR: integrated stress response
LHON: Leber’s Hereditary Optic Neuropathy
MAM: mitochondria-associated membrane
MEF: mouse embryonic fibroblast
MELAS: mitochondrial myopathy, Encephalopathy, Lactic Acidosis, Stroke-like episodes
MERRF: myoclonic epilepsy with ragged red fibers
mETC: mitochondrial electron transport chain
MRS: mitochondrial retrograde signaling
MURE: mitochondrial UPR response element
NARP: neurogenic ataxia and retinitis pigmentosa
OxPhos: oxidative phosphorylation
PEO: progressive external ophthalmoplegia
PERK: PKR-like endoplasmic reticulum kinase
PGC1α: PPARγ coactivator 1α
PKR: dsRNA-activated protein kinase
ROS: reactive oxygen species
TFAM: mitochondrial transcription factor A
TOR: target of rapamycin
Trib3: tribbles homolog 3
UCP1: uncoupling protein 1
uORF: upstream open reading frames
UPR: unfolded protein response
